# The Neurovascular Unit: Effects of Brain Insults During the Perinatal Period

**DOI:** 10.3389/fnins.2019.01452

**Published:** 2020-01-22

**Authors:** Alexander H. Bell, Suzanne L. Miller, Margie Castillo-Melendez, Atul Malhotra

**Affiliations:** ^1^Department of Paediatrics, Monash University, Melbourne, VIC, Australia; ^2^The Ritchie Centre, Hudson Institute of Medical Research, Melbourne, VIC, Australia; ^3^Department of Obstetrics and Gynaecology, Monash University, Melbourne, VIC, Australia; ^4^Monash Newborn, Monash Children’s Hospital, Melbourne, VIC, Australia

**Keywords:** blood–brain barrier, hypoxia, hypoxia-ischemia, intrauterine growth restriction, prematurity, astrocytes, pericytes, basement membrane

## Abstract

The neurovascular unit (NVU) is a relatively recent concept in neuroscience that broadly describes the relationship between brain cells and their blood vessels. The NVU incorporates cellular and extracellular components involved in regulating cerebral blood flow and blood–brain barrier function. The NVU within the adult brain has attracted strong research interest and its structure and function is well described, however, the NVU in the developing brain over the fetal and neonatal period remains much less well known. One area of particular interest in perinatal brain development is the impact of known neuropathological insults on the NVU. The aim of this review is to synthesize existing literature to describe structure and function of the NVU in the developing brain, with a particular emphasis on exploring the effects of perinatal insults. Accordingly, a brief overview of NVU components and function is provided, before discussion of NVU development and how this may be affected by perinatal pathologies. We have focused this discussion around three common perinatal insults: prematurity, acute hypoxia, and chronic hypoxia. A greater understanding of processes affecting the NVU in the perinatal period may enable application of targeted therapies, as well as providing a useful basis for research as it expands further into this area.

## Introduction

The neurovascular unit (NVU) is a relatively recent concept in neuroscience, representing the structural and functional multicellular relationship between the brain and blood vessels. The cellular components are the neurons, perivascular astrocytes, microglia, pericytes, endothelial cells (EC), and the basement membrane (BM). The components of the NVU share intimate and complex associations, and it is these associations that have led to their classification as a single functioning unit. The NVU is responsible for the maintenance of a highly selective blood–brain barrier (BBB) and cerebral homeostasis, as well as the control of cerebral blood flow (CBF) (Abbott et al., 2006). Since its genesis in 2001 (Iadecola, 2017), an increasingly large volume of literature has been produced on the NVU across a relatively short period, which has allowed our understanding to develop quickly across the same timeframe. Currently, the building blocks and the phenotype of the NVU are well established, and signaling between the different components has received considerable attention in adult disease. However, relatively little research has looked specifically at the NVU in the developing brain and perinatal period. This has meant that despite the impressive pace at which our understanding of the NVU has advanced, there remain significant gaps in our knowledge of NVU development, and the role the NVU plays in the developing brain.

One area of particular interest when it comes to perinatal brain development is the impact of known conditions and insults commonly affecting the newborn brain on the NVU during this period, and the role of the NVU in mediating brain injury and regeneration. Despite a relative lack of research conducted with the specific objective of determining these impacts, there nonetheless exist a variety of studies measuring outcomes that can be used to infer such effects. The aim of this review is to synthesize existing literature on the NVU in the developing brain, exploring in particular the effects of perinatal insults. A brief overview of NVU components and function is provided, before discussion of NVU development and how this may be affected by perinatal pathologies. This discussion will be based specifically around three common perinatal insults: prematurity, acute hypoxia, and chronic hypoxia. A greater understanding of processes affecting the perinatal NVU may enable application of targeted therapies, as well as being useful as a basis for research as it expands further into this area.

## NVU Function

The NVU plays a variety of roles within the brain, although two processes in particular display particularly intimate involvement. Neurovascular coupling (NVC), often referred to as functional hyperemia, is the mechanism by which local blood supply is matched to neuronal demand via changes in vascular intraluminal diameter (Carmignoto and Gomez-Gonzalo, 2010). The NVU is the fundamental driver of this process, providing the basis for linking neurons to cerebral vessels (Muoio et al., 2014). NVC mechanisms are thought to be initiated via glutamate, released from activated neurons (Girouard and Iadecola, 2006). Glutamate then activates astrocytes and pericytes, along with other neurons, inducing the release of vasoactive mediators from these cells. The balance of vasoconstrictive and vasodilatory mediators controls the tone of the surrounding vasculature, regulating local CBF (Hendrikx et al., 2019).

Barrier function is the second major process that is enabled by the NVU. The perinatal BBB is reviewed excellently by Saunders et al. (2018) alongside other barrier mechanisms in the developing brain, including the blood-cerebrospinal fluid barrier. In contrast to the BBB, the blood-cerebrospinal fluid barrier is not a function of the NVU, with epithelial cells of the choroid plexus playing the major cellular role (Saunders et al., 2018). Conversely, NVU component cells play an integral role in all stages of BBB development and maintenance, providing a barrier that is fundamental in maintaining an optimal environment for brain function (Sa-Pereira et al., 2012). ECs provide the major cellular contribution to the BBB, largely through the physical barrier provided to paracellular permeability by inter-endothelial tight junctions (Zlokovic, 2008). ECs also express receptors and ion channels involved in barrier function, and contribute to homeostatic BBB roles through enzymatic metabolism (Persidsky et al., 2006). Evidence also supports astrocyte, pericyte, and neuronal involvement in BBB formation and maintenance, with dysfunction or aberrant activation in these cell types often resulting in BBB impairment (Persidsky et al., 2006).

## Components of the NVU in the Adult Brain

**FIGURE 1:**
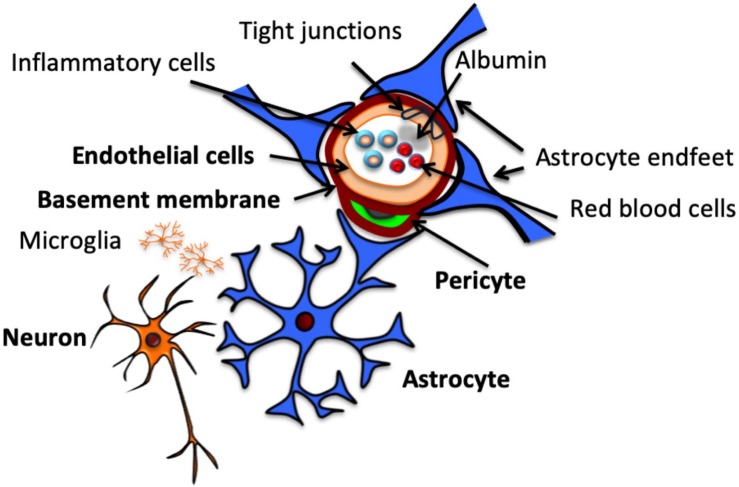
Structure of the neurovascular unit. Schematic representation of NVU structure, including neurons, which communicate with the astrocytes that surround the vasculature by extending specialized end feet. Pericytes also extend around the vasculature, sitting between end feet and endothelial cells, which make up the vascular wall, encased by a continuous basement membrane. Endothelial cells are connected by tight junctions, which contribute to BBB function by preventing paracellular transport of intraluminal substances, including cells and proteins.

An exploration of the effects of perinatal insults on the NVU would be incomplete without first considering the individual cellular elements that make up this complex in the developing brain. These elements and their topographical arrangement are presented in Figure 1. Components of the NVU interact with each other, each making its own contribution to the overall function of the structure. An understanding of these components provides essential context for considering how they are impacted in the presence of pathological insults. Presented in this review is a brief description of the major NVU component cells. A comprehensive overview of these components and their roles in the adult NVU can be found in reviews by Muoio et al. (2014) and McConnell et al. (2017).

### Neurons

The role of neurons has been described as that of a “pacemaker” within the NVU due to their role in regulating CBF (Muoio et al., 2014), and they play a fundamental regulatory role in NVC (Petzold and Murthy, 2011). Neurons are particularly sensitive to small changes in oxygen and nutrients carried by the blood, generating signals in response to these. These signals alert nearby astrocytes, either directly or through interneurons, resulting in compensatory mechanisms being activated at the level of the vasculature (Figley and Stroman, 2011; Muoio et al., 2014). Neurons also play an important role in cerebrovascular organization, with neural activity reflecting the extent of vascular development and density in the brain (Lacoste et al., 2014). The intimate relationship between neurons and other cells becomes especially important in developing brain, where a critical window for neuronal stimulus of angiogenesis has been observed in mouse models during the neonatal period. Within this window, chronic or pathological neuronal stimulation leads to arrested angiogenesis in the associated brain area, which persists well beyond cessation of the stimulus (Whiteus et al., 2014). This effect is not replicated in the adult brain (Whiteus et al., 2014), highlighting the unique nature of the perinatal brain and the need for a specific understanding of its development and response to stimuli.

### Astrocytes

Astrocytes sit between neurons and ECs within the NVU (Figure 1; Zonta et al., 2002), acting as conduit cells between the two structures (Petzold and Murthy, 2011). NVU astrocytes extend “end-feet” processes from cell bodies to surround the arterioles and capillaries. These astrocytic end-feet provide almost complete coverage of the cerebral vasculature (Mathiisen et al., 2010), facilitating bidirectional communication between astrocytes and ECs (Gordon et al., 2011).

The traditional understanding of the role of perivascular astrocytes has largely centered on their contribution to the BBB (Arthur et al., 1987; Sofroniew and Vinters, 2010). Strong *in vitro* evidence points to astrocytic involvement in upregulating mechanisms involved in several BBB features (Abbott et al., 2006), but has particularly concentrated on the essential role of astrocytes initiating formation of the BBB (Arthur et al., 1987). This role has not gone unchallenged, however, with a point of contention the appearance of astrocytes after the initial commitment to barrier formation in capillaries (Holash et al., 1993). Astrocytic involvement in the BBB extends beyond development and structure, with roles for astrocytes also implicated in homeostatic BBB mechanisms. An abundance of aquaporin 4 water channels and K^+^ transporters in astrocyte processes make them specially adapted for recycling of ions and neurotransmitters, as well as removal and excretion of water from the brain (Abbott et al., 2006; Sofroniew and Vinters, 2010). These functions are essential to the brain’s ongoing health and underscore the importance of astrocytes within the BBB, continuing into adulthood.

More recently, interest in perivascular astrocytes has extended to their role in NVC, and subsequent control of CBF. It is now known that astrocytes mediate both dilation and constriction of cerebral blood vessels (Metea and Newman, 2006). Astrocytes respond to neurotransmitters released at synapses of adjacent neurons (Gordon et al., 2007), with glutamate the only neurotransmitter definitively implicated in NVC (Petzold and Murthy, 2011). Glutamate release induces an increase in astrocytic intracellular free Ca^2+^ (Cornell-Bell et al., 1990), which Zonta et al. (2002) proposed prompts the release of a vasoactive arachidonic acid metabolite from astrocyte end-feet. With the support of subsequent research (Takano et al., 2006), the involvement of astrocytic arachidonic acid metabolite in vascular modulation is now widely accepted. Astrocyte-mediated vasoconstriction was discovered after this vasodilatory process (Mulligan and MacVicar, 2004), adding a layer of complexity to our understanding of the role of astrocytes in NVC. The finding initially challenged the demonstrated mechanism of astrocyte-mediated vasodilation, with vasoconstriction also involving astrocytic increases in free Ca^2+^ (Mulligan and MacVicar, 2004). This apparent paradox was subsequently resolved when it was found that astrocyte responses to raised Ca^2+^ were dependent on the metabolic environment, with lower O_2_ availability favoring vasodilation (Gordon et al., 2008). Evidence has also supported other mechanisms to explain this contradiction, including roles for the magnitude of astrocytic Ca^2+^ increase (Girouard et al., 2010), resting vascular tone (Blanco et al., 2008), and enzymatic inhibition by nitric oxide (Metea and Newman, 2006).

### Pericytes

Described for many years as support cells with a limited role in neurovascular functioning, like astrocytes much has been established in recent years regarding the important and diverse roles that pericytes play as NVU components. Pericytes extend along the microvasculature in every capillary within the brain, making direct contact with the underlying endothelium and embedded within the vascular BM. They play a variety of important roles in the development, maturation, and functionality of microvascular networks (Balabanov and Dore-Duffy, 1998). Though various important roles for NVU pericytes have been documented, a major function appears to relate to their role in BBB development and function (Sa-Pereira et al., 2012). The extent of pericyte coverage of ECs throughout the body appears to be correlated to the integrity of the vascular barrier these ECs provide, with CNS vasculature recording the greatest pericyte coverage in the body (Shepro and Morel, 1993; Daneman et al., 2010). At the BBB, pericytes exert their influence in at least two major ways. Firstly, they regulate gene expression within neighboring ECs via upregulation of the production of certain markers associated with BBB function, and secondly by mediating the polarization and attachment of astrocyte end-feet to blood vessels (Armulik et al., 2010).

In addition to their importance in barrier function, pericytes are also among the coordinated elements that combine to regulate NVC. Through their unique position surrounding the ECs of the vascular wall, pericytes are able to use changes in cell length to influence vascular diameter, and therefore local blood flow (Sa-Pereira et al., 2012; McConnell et al., 2017). Pericytes express a number of contractile proteins, including α-smooth muscle actin, tropomyosin, and myosin, as well as receptors for vasoactive peptides (Sa-Pereira et al., 2012). Pericytes (and myocytes in larger vessels) have a basal level of contractility that is expressed under normal physiological conditions when oxygen and nutrients are in good supply. This basal contractility is the result of the contractile proteins present in pericytes and represents the product of a balance of signals initiated by neurons, which act via the vasoactive receptors present (Figley and Stroman, 2011; Sa-Pereira et al., 2012). Changes in these neuronal signals alter pericyte contractility, and it is via these changes that pericytes are responsible for the changes in intraluminal diameter that form the basis for the vascular aspect of the NVC response.

### Endothelial Cells

Endothelial cells line the cerebral vasculature and provide the major anatomical BBB contribution (McConnell et al., 2017). Although previously thought of as passive NVU constituents (Muoio et al., 2014), it is now known that ECs play an active role in a number of NVU processes. ECs of the cerebral microvasculature are specifically adapted to these roles, displaying characteristics that make them unique to other ECs throughout the body (Sa-Pereira et al., 2012) such as reduced wall thickness, and lack of fenestrations (Stamatovic et al., 2008).

The major contribution ECs provide to the BBB is conferred through the presence of TJs between adjacent cells, which provide a physical barrier preventing paracellular diffusion of polar blood solutes (McConnell et al., 2017). Endothelial TJs are generated through a backbone of three major transmembrane proteins and accompanying membrane-associated cytoplasmic proteins (Luissint et al., 2012).

Among transmembrane proteins involved in TJ function are a family of proteins called occludins, although the extent of their contribution to TJs is controversial. Evidence has suggested a key role for them in TJs (Balda, 1996; Xu et al., 2012), however, this has been contradicted by research demonstrating an ability for functional TJs to exist without occludin (Saitou et al., 1998). Crucially, most research suggesting occludin is non-essential has focussed on epithelial TJs, meaning it remains possible that its necessity is limited to TJs between brain ECs.

Another family of transmembrane proteins with a demonstrated involvement in endothelial TJs are claudins. The contribution of claudins to endothelial TJs is far less controversial than that of occludins, and they are acknowledged as playing a key role in TJ formation and integrity (Luissint et al., 2012). Demonstrating this role, rat brain ECs display increased barrier function with exogenous expression of a claudin subtype (Ohtsuki et al., 2007), whilst mice deficient in the same claudin subtype display increased BBB permeability (Nitta et al., 2003).

The third identified family of transmembrane proteins comprising the endothelial TJ backbone are junctional adhesion molecules. Junctional adhesion molecules do not have an essential role in TJ formation, however, may be involved in assembling TJ components and establishing cell polarity (Luissint et al., 2012). The endothelial cytoskeleton further reinforces TJ integrity through attachments made with the transmembrane/cytoplasmic protein backbone (Stamatovic et al., 2008).

Though important, TJs do not provide the only endothelial contribution to the BBB. ECs display a polarity between biochemical and functional properties of their luminal and antiluminal membranes, including expression of metabolic enzymes, receptors, ion channels, and transporters (Betz et al., 1980; McConnell et al., 2017). This allows ECs to function as a selective transport interface, ensuring brain tissue can receive required nutrients, and excrete metabolic waste products (Stamatovic et al., 2008).

Endothelial transporters are highly selective, playing a crucial role in brain homeostasis by ensuring that large and polar compounds that would otherwise be unable to cross the BBB are able to do so where necessary (Ghersi-Egea et al., 2018). Transporters provide mechanisms for both influx of beneficial compounds required within the brain, and efflux of potentially harmful compounds out of the brain (Saunders et al., 2018). Major endothelial transporter groups at the brain include hexose transporters, such as GLUT1 (Patching, 2017), and amino acid transporters, such as LAT1 and LAT2 (Kido et al., 2001), among others (Pardridge, 1995). Endothelial transporters at the BBB contribute further to brain homeostasis via their dynamic regulation. Evidence has been found for changes in the expression and function of a range of transporters at the in response to various factors, including neurotransmitter levels, cytokines, and physiological states such as starvation and sleep deprivation (Kastin and Pan, 2000; Avemary et al., 2013; Keaney and Campbell, 2015).

Although most current knowledge of ECs as a component of the NVU surrounds their role within the BBB, it is thought that they may be involved in other NVU processes, such as NVC. Current research into a potential EC role in NVC is limited, however, it has been noted that they produce several vasoactive products, both dilatory and constrictory (Muoio et al., 2014). Given our relative lack of knowledge of the mechanisms facilitating NVC, the existence of such products provides a solid basis for further investigation into a role for ECs in the process.

### Basement Membrane

Also known as the basal lamina, the BM represents an extracellular matrix structure, formed from proteins thought to be secreted by ECs and pericytes (Stratman and Davis, 2012). The BM surrounds the EC layer of capillaries, separating it from surrounding pericytes and astrocyte end-feet, as well as duplicating around pericytes to separate them from astrocytes (Balabanov and Dore-Duffy, 1998). The complexity of the BM has seen it often ignored in favor of cellular NVU components (Gautam et al., 2016), however, this belies the important contribution it makes. The BM plays a vital role in vascular integrity, providing anchoring support to vessels and surrounding cells (Yurchenco and Patton, 2009). These cells express adhesion receptors for BM proteins at their cell surfaces, permitting close associations and structural connection (Yurchenco and Patton, 2009).

Although the BM does not mediate any significant barrier function of its own (Sa-Pereira et al., 2012), it is crucial to the function and maintenance of the BBB. BM disorder can cause disruption of TJ proteins through effects on EC cytoskeletons, which in turn leads to BBB compromise (Cardoso et al., 2010). Changes in BM protein expression are also seen following several pathological insults, including ischemia, and are associated with reduced barrier integrity and edema (Del Zoppo et al., 2006). These demonstrations of BM function help to illuminate its importance as a component of the NVU.

## NVU in the Perinatal Brain

Despite extensive research into the mature NVU since the widespread embrace of the concept, there has to date been relatively little research performed on the NVU within the developing brain. Illustrating this, in a review of the developing BBB, Saunders et al. observed a widespread characterization of the BBB as being “immature”, noting that this was often a vague description based on minimal evidence (Saunders et al., 2012).

In reality, little is known about the NVU in the perinatal period, however, evidence points to considerable differences between the perinatal NVU and the adult NVU. BBB function in newborns differs substantially from that of adults in both physiological and pathological circumstances (Braun et al., 1980; Anthony, 1997; Fernandez-Lopez et al., 2012). In addition, Kozberg and Hillman (2016) discuss the NVC in the perinatal brain, highlighting the ways that this coupling function also differs from its adult equivalent. Combined, these differences suggest a disparity in NVU structure and/or function between the developing and adult brain. This disparity highlights the dangers of relying on knowledge of adult brain structure and function to inform our approach to the developing NVU, emphasizing the importance of research specifically looking at the NVU in the perinatal period.

### Perinatal Brain Development

Current evidence suggests that NVU development is staggered, with different components emerging and maturing at different points. Much knowledge of NVU development has been gathered from studies in rodents. In developing mice, vascular invasion of the neuroepithelium has been observed at embryonic day 9–10 (E9–10) (Bauer et al., 1993), where the mouse nervous system is approximately equivalent to human embryonic day 26 (Otis and Brent, 1954). Alongside these invading ECs were pericyte-like cells, suggesting pericytes appear in the brain during early vascular development (Bauer et al., 1993). The early appearance of pericytes in embryogenesis is consistent with observations suggesting they play a key role in cerebral angiogenesis. Pericytes generate early microvascular structures before recruiting ECs to line these vessels through secretion of factors such as vascular endothelial growth factor (VEGF) (Virgintino et al., 2007).

In contrast to the early appearance of pericytes, astrocyte involvement in the NVU appears to begin much later in development. In rats, the first signs of astrocyte presence are observed around birth, having developed from precursor radial glia cells, and by around 2 weeks post-delivery end-feet have completed coverage of the CNS vasculature (Daneman et al., 2010; Rowitch and Kriegstein, 2010). Interestingly, astrocytes appear earlier in humans, with end-feet coverage of vasculature completed in the second half of gestation (El-Khoury et al., 2006). Although this suggests a more integrated developmental role, it is more likely to reflect altered developmental timelines between the two species. Using a detailed model, Clancy et al. (2001) equates cortical development in rats at birth and postnatal day 14 (P14) to the human cortex at approximately 16 and 29 weeks’ gestation, respectively. Not only does this timeline place astrocyte development in humans and rats within roughly the same stage of cortical development, it also serves to reinforce some of the limitations imposed by relying on animal models to inform our knowledge of NVU development.

The timing of BBB formation has provided a point of contention in the past. As discussed, Saunders et al. (2012) made note of the commonly held, though largely unfounded, belief that the BBB within the developing brain was “immature” and lacked full functionality. Mallard et al. (2018) have devoted a review to challenging this belief even further, highlighting a variety of neurodevelopmental functions contributed to by the BBB and other barrier mechanisms in fetal and newborn brains. One recent investigation has identified that BBB function is established prior to birth, although at different developmental points for different brain areas (Ben-Zvi et al., 2014). This study measured BBB function using a technique involving a tracer injected into the embryonic liver to circumvent the artificial leakiness phenotypes the authors claimed more traditional *trans*-cardiac tracer perfusion could cause, due to changes in blood pressure. Although the evidence for these claims is unsubstantial, and injection of tracer into the embryonic liver represented a novel and unvalidated technique, the finding of a functional BBB prenatally is supported elsewhere by immunohistochemical staining in the embryonic rat brain showing confinement of plasma proteins to the vasculature (Saunders and Habgood, 2011; Saunders et al., 2012).

Studying developing rats in an attempt to profile BBB development, Daneman et al. (2010) demonstrated EC expression of tight junction proteins and BBB transporters at E12, around the beginning of cerebral angiogenesis. They also conducted functional testing using a molecular tracer from E15 to P20, demonstrating a functional BBB across all time points (Daneman et al., 2010). Although this confirms the early establishment of BBB function during embryogenesis, research using electrical resistance to measure BBB permeability in pial vessels shows a progressive increase in function during fetal development, up to birth (Butt et al., 1990). This suggests BBB function continues to develop across gestation, although the generalizability of patterns in the BBB function of pial vessels to the smaller intracerebral vessels of the NVU remains unknown. Of particular note in studies of the developing BBB is the existence of a functional barrier prior to astrocyte appearance, suggesting other agents may contribute the previously discussed role of astrocytes in the mature BBB during fetal development. These combined observations from rodents suggest a developmental pattern whereby NVU components associate from early embryogenesis, producing a functional BBB. These components and associations subsequently continue to develop and increase the integrity of the barrier. Such a pattern is consistent with the timeline for murine BBB development proposed by Zhao et al. (2015) which placed barrier genesis as beginning around E15, and continuing through to the postnatal period. According to this timeline, BBB components such as pericytes, BM, transporters, and TJs all appear slightly earlier in the embryonic period. Although this reinforces their essential contributions to barrier function, it is important to note that each of these components may develop at distinct rates, and as such their influence on different stages of barrier genesis may also differ. For example, although the endothelial transporter GLUT1 appears in the embryonic stage, levels have been found to be low in the newborn rat brain, before doubling between P14–21, and again between P21–30 (Vannucci, 1994).

It is important to note that although rodents provide much of our current knowledge of NVU development, there are several important known differences between cerebrovascular development in humans and rodents. The aforementioned difference in timelines for astrocyte appearance demonstrates just one example of the disparities that mean animal models can be relied on only as a guide. Indeed, Dobbing and Sands (1979) present a detailed discussion of brain growth patterns among several mammals, highlighting significant differences between human and rodent developmental trajectories. Among findings presented is a comparison of relative brain development at birth, which places the human birth brain weight at 27% of the weight of a fully grown adult brain, compared to an equivalent figure in rats of 12% (Dobbing and Sands, 1979). Thus, although rodent research has enhanced our understanding of NVU development, we must also recognize the limitations of generalizing the results to humans and maintain caution in how we apply them.

### Pathophysiology

Although our knowledge of perinatal NVU development remains incomplete, there is substantial evidence suggesting that pathophysiological states during the perinatal period can affect NVU structure and function. Antenatally for example, it has been suggested that uteroplacental inflammation, such as is caused by uteroplacental infection, can result in BBB compromise, reflecting NVU dysfunction (Hutton et al., 2007). This is largely based on research documenting raised albumin immunoreactivity in the cerebellar parenchyma following uteroplacental inflammation, although the degree to which this is caused by BBB dysfunction remains unclear. The largely intracellular localization of immunoreactivity, including within Purkinje cells, makes it possible that the increase may reflect other mechanisms, such as the uptake of proteins from CSF previously documented in Purkinje cells (Borges et al., 1985; Fishman et al., 1990). Postnatally, insults as diverse as neonatal seizures and certain surgical procedures have also been associated with structural and functional disruptions to components of the NVU. Neonatal seizures affect both BBB integrity and CBF, likely reflecting disturbances in several NVU components (Zimmermann et al., 2008). Surgical procedures such as cardiopulmonary bypass (CPB) have also demonstrated potential to cause extensive neonatal BBB compromise, increasing with duration of CPB (Cavaglia et al., 2004). Such compromise is not seen in adult brain tissue (Laursen et al., 1986; Gillinov et al., 1992; Waaben et al., 1994), suggesting an enhanced vulnerability to insults in the developing brain.

**FIGURE 2:**
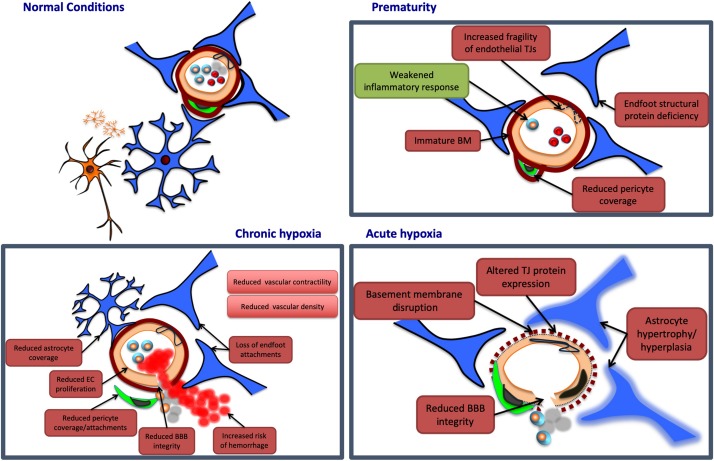
Potential neurovascular unit responses to perinatal insults. Pathological insults during the perinatal period may lead to alterations in NVU structure and/or function. Potential damaging alterations are presented in red, while protective effects are presented in green. In prematurity, the cerebrovasculature of certain regions may display an immaturity in the structure of the basement membrane (BM), and endothelial tight junctions (TJs), potentially leading to increased fragility in these vessels. Similarly, premature birth may be associated with a deficiency in certain structural proteins of astrocyte end feet, and with a reduction in vascular coverage by pericytes, contributing to further NVU dysfunction. Prematurity may also provide protection at the NVU, however, by weakening otherwise damaging inflammatory responses. Acute hypoxia may induce NVU dysfunction by disrupting the basement membrane, and altering endothelial TJ protein expression, both of which may reduce blood–brain barrier (BBB) integrity. Acute hypoxic insults are also associated with hypertrophy and hyperplasia of astrocytes, persisting for weeks after the initial injury. Chronic hypoxia has been associated with reductions in endothelial cell (EC) proliferation, as well as coverage and attachments of both astrocytes and pericytes. These associations may contribute to a reduction in BBB integrity, and an increased risk of vascular hemorrhage. Chronic hypoxic states have also been linked to reductions in vascular contractility and density in various brain regions.

Most current research into perinatal NVU pathology has examined insults falling within three broad pathological processes: prematurity, acute hypoxia, and chronic hypoxia (Figure 2). The interest in these states reflects their prevalence, as well as the variety of outcomes they are likely to impact on structure and function. Although here we consider each of these processes individually, in both research and clinical practice they often exist simultaneously. Where hypoxic and premature insults have been studied alongside one another, we have included the research under the relevant hypoxia heading. In doing so, we have allowed for particular attention to be paid to any changes in the NVU response to premature birth resulting from the addition of hypoxic insult.

#### Prematurity

Premature birth in humans is defined as any delivery occurring before 37 weeks of gestation (Engle, 2006). Prematurity is a major area of concern in public health, being associated with substantial morbidity and mortality both in the newborn period and in later life (McCormick et al., 2011).

Preterm birth is an area of significant interest in the study of the perinatal NVU (see Table 1). Cerebral neuropathologies are often observed in infants born preterm, particularly with increasing degree of prematurity, and white matter injury is the most common neuropathology in this cohort of infants (Rees and Inder, 2005). Gilles et al. (2018) posited seven factors underlying the association between neurological deficit and preterm birth: highly active developmental processes, lack of essential long chain fatty acids or fatty acid transporters, inability to synthesize sufficient growth factors for development, insufficient growth factors for protection, harmful exposures around delivery, sustained, excessive inflammation due to an immature immune system, and damage to the BBB due to inflammation. Although a great deal of uncertainty remains around NVU changes in premature infants, available evidence suggests that prematurity may be associated with significant NVU compromise.

**TABLE 1:** Studies investigating the effects of prematurity on the NVU.

**Authors**	**Experimental model**	**NVU outcome measured**	**Key findings**
Sotrel and Lorenzo, 1989	E28 rabbits (term = ∼32 days)	GM vessel morphology	In GMH, narrow and wide gaps between intact endothelial cells existed, filled with luminal contents; BM of GM vessels was thin, poorly defined, and often discontinuous, contrasting the developed BM of other brain areas; cells appearing to be poorly defined astrocytes sat adjacent to GM vessels, leaving the vessel wall ∼40% uncovered
Ment et al., 1995	Newborn beagle pups (GM comparable to human preterm neonate) at P1, 4, and 10	TJ length, BM area, percentage coverage of vessel walls by supporting cells; outcomes measured in both GM and PVWM	GM BM area increased significantly between P1–4, remaining increased to P10; PVWM BM area showed no significant change from P1–10; GM TJ length did not increase significantly from P1–4, but showed significant increase from P4–10; PVWM TJ length showed no significant change from P1–10; percentage coverage of GM vessel walls by supporting cells did not increase significantly from P1–4, but showed significant increase from P4–10; PVWM vessel wall coverage showed no significant change from P1–10
El-Khoury et al., 2006	Human autopsy brain samples from premature infants of 23–40 weeks gestation	Astrocyte end feet vessel coverage; coverage was compared between GM, WM, and cerebral cortex	AQP4+ end feet developed earlier in gestation than GFAP+ end feet in all areas observed; GFAP+ end feet coverage was lower in GM than other areas from 23 to 34 weeks gestation, with no difference in AQP4+ end feet coverage between areas
Ballabh et al., 2007	Human autopsy brain samples from 23 to 40 weeks gestation; brain samples from spontaneous abortuses of 16–22 weeks gestation	Endothelial proliferation; proliferation was compared between the GM, and cortex and WM regions	EC proliferation was significantly greater in the germinal matrix than the cortex or WM in both fetuses and premature infants; GM EC proliferation was greater in fetuses than premature infants
Braun et al., 2007	Human autopsy brain samples from 23 to 40 weeks gestation; E29 rabbits (term = ∼32 days)	Pericyte coverage and density; coverage was compared between the GM and adjacent WM and cortex	Both pericyte coverage and density were significantly lower in the GM than in the cortex or WM for all gestational age categories using IHC; ultrastructural analysis showed significantly reduced pericyte numbers in prematurity in both human and rabbit GM, compared with cortex and WM; suppression of VEGF significantly enhanced GM pericyte coverage, although it remained reduced compared with other regions
Brochu et al., 2011	Naturally delivered P1 and P12 rats (equivalent to human preterm and term newborns, respectively, in terms of cerebral development); some rats were injected with LPS alone, while some had HI induced without LPS injection, and some rats were injected with LPS and subsequently had HI induced; brain tissue collected at 4, 24, 48 h and 8 days post-HI	BBB permeability	Exposure to HI injury with/without LPS led to increased BBB permeability at P12, but not at P1, at 48 h post-HI; no change in BBB permeability detected before 48 h post-HI in any experimental condition

*AQP4, aquaporin-4; BBB, blood–brain barrier; BM, basement membrane; E(28), embryonic day (28); EC, endothelial cell; GFAP, glial fibrillary acidic protein; GM, germinal matrix; GMH, germinal matrix hemorrhage; HI, hypoxia-ischemia; IHC, immunohistochemistry; LPS, lipopolysaccharide; NG2, Neuron glia antigen-2; P(1), postnatal day (1); PDGFR-β, platelet derived growth factor receptor beta; PVWM, periventricular white matter; TJ, tight junction; VEGF, vascular endothelial growth factor; WM, white matter.*

One association of prematurity that almost certainly involves NVU dysfunction is germinal matrix hemorrhage (GMH), a disorder carrying potentially devastating sequelae including cerebral palsy, post-hemorrhagic hydrocephalus, and severe cognitive impairment (Lekic et al., 2012). GMH is the most common cause of intraventricular hemorrhage (IVH) (Strahle et al., 2012) and occurs more frequently in premature infants delivered during the high risk period of gestational age 24–32 weeks (Ment et al., 1995). Interestingly, some evidence suggests GMH incidence is independent of gestational age within this risk period (Ment et al., 1995), although this is challenged by other research suggesting that risk is highest among premature infants delivered prior to 29 weeks (Dolfin et al., 1983).

Several hypotheses for increased rates of GMH in prematurity have been proposed, which implicates a role for the NVU and its components. It has been suggested that immaturity of the surrounding NVU components during the GMH risk period prevents appropriate development of endothelial TJs and the BM in this region, resulting in a relative inability for vessels to withstand increases in pressure or other insults (Sotrel and Lorenzo, 1989). Similarly, a relative deficiency in expression of crucial structural proteins of astrocyte end-feet has been found in the germinal matrix compared with other brain regions during the GMH risk period (El-Khoury et al., 2006). This could present a cytostructural weakness that may be responsible for increased fragility of germinal matrix vessels within this period.

More recently, it has been noted that the germinal matrix displays a lower density and coverage of pericytes than other brain regions in premature infants, an observation made using both immunohistochemistry and electron microscopy (Braun et al., 2007). Given the important role of pericytes in cerebral vascular stability, a relative deficiency of pericytes in the germinal matrix of preterm infants could underlie vascular instability, and therefore this could present a mechanism for the increased rate of hemorrhage observed. A potential protective role for pericytes in GMH is further suggested by findings that inhibiting angiogenesis in the premature germinal matrix results in both increased pericyte coverage (Braun et al., 2007), and decreased GMH incidence (Ballabh et al., 2007).

Although we speculate that increased GMH incidence in preterm infants reflects potential NVU dysfunction, it is important to note that the NVU may conversely mediate greater protective benefit in the preterm brain. Perinatal brain inflammation induced in rat models through exposure to lipopolysaccharide and/or hypoxia-ischemia showed a number of differences in outcomes between preterm- and term-equivalent newborns. Among these were distinct patterns of neuroinflammatory response between the two groups, wherein the authors noted BBB disruption in term brains, but not in preterm brains (Brochu et al., 2011). Importantly, these observations of disruption were based on immunostaining for albumin, where staining distribution and intensity was observed but not quantified. Thus, although suggesting incomplete development of certain processes in premature infants may in fact protect the NVU by preventing otherwise damaging mechanisms, it is important that such results are built upon with more thorough research to investigate these potential effects and their mechanisms.

#### Acute Hypoxia

Given the prevalence of perinatal exposure to hypoxia and its documented effects on the brain (Douglas-Escobar and Weiss, 2015), it is unsurprising that the impact of hypoxic insults is among the most widely studied areas of perinatal NVU pathology (see Table 2). There are several potential causes of acute hypoxia in the perinatal brain, with hypoxic-ischemic (HI) injury among the most widely studied. This type of injury is widely considered to be responsible for most instances of brain injury arising from prematurity, though this link has been strongly questioned in recent years (Gilles et al., 2018). Although definitions and underlying causes can be vague, HI injury primarily refers to neuropathology attributable to impaired CBF (Perlman, 2006). Most commonly, this occurs perinatally as a result of interrupted blood flow and gas exchange at the placenta, and thus represents an acute event (Perlman, 2006). The basis for neurologic damage caused by HI injury is thought to largely involve hypoxia, induced by either hypoxemia or ischemia (Rivkin and Volpe, 1993; Perlman, 2006), which results in a multi-phase process of complex biochemical interactions. This process is characterized by initial deleterious mechanisms in response to hypoxia, known as “primary energy failure”, before a latent period of 6–24 h during which blood flow is restored and reperfusion occurs. Following this latent period further deleterious mechanisms commence in a phase known as “secondary energy failure” (Gopagondanahalli et al., 2016). In severe cases HI injury may progress to hypoxic-ischemic encephalopathy (HIE), which in contrast to HI injury is diagnosed using objective clinical criteria (Douglas-Escobar and Weiss, 2015; Gopagondanahalli et al., 2016).

**TABLE 2:** Studies investigating the effects of acute hypoxia in the perinatal period on the NVU.

**Authors**	**Experimental model**	**NVU outcome measured**	**Key findings**
Vannucci et al., 1996	P7 rats; unilateral HI brain injury induced followed by immersion in warm water bath, then temporary exposure to hypoxia; brain tissue collected at 4, 24, and 72 h post-HI	Endothelial GLUT1 transporter expression	Slightly increased bilateral GLUT1 expression at 4 h; substantially increased ipsilateral expression at 24 h, with contralateral expression returning to control levels; no significant difference at 72 h between bilateral expression and control levels
Muramatsu et al., 1997	P7, P14, P21 rats; unilateral HI brain injury induced followed by temporary exposure to hypoxia; brain tissue collected at 3, 6, 9, 12, 18, and 24 h post-HI	BBB permeability	Increased BBB permeability within 6 h of HI injury in P7 rats, and within 12 h in P14 rats; little-to-no increase in BBB permeability in P21 rats up to 24 h
Levison et al., 2001	P7 rats; unilateral HI brain injury induced followed by temporary exposure to hypoxia; brain tissue collected at 21 days	Astrocyte morphology	Astrocyte hyperplasia and hypertrophy found throughout the brain; astrocytes found to have replaced other cells in some regions
Malaeb et al., 2007	E112–117 sheep (term = ∼145 days); bilateral HI injury induced, followed by 72 h reperfusion	TJ protein expression	HI-reperfusion led to increased claudin 5, and decreased ZO-1 and ZO-2 expression
Svedin et al., 2007	P9 MMP-9 knockout mice; moderate or severe unilateral HI injury induced followed by temporary exposure to hypoxia; brain tissue collected at 0, 1, 3, 6, 24 and 72 h post-HI	BBB permeability	Increased BBB permeability from 3 to 72 h following severe HI, with highest permeability 24 h after HI; Increased BBB permeability from 3 to 72 h in WT mice following moderate HI, but only at 6 and 24 h in MMP-9 KO mice
Kumar et al., 2008	Term human neonates with perinatal asphyxia and subsequent HIE; participants at 12–24 h of life	BBB permeability	BBB permeability increased significantly with progression of HIE
Chen et al., 2012	E127 sheep; bilateral HI brain injury induced, followed by reperfusion for 4, 24, or 48 h	BBB permeability; endothelial TJ protein expression	Permeability was highest after 4 h reperfusion, compared with 24 and 48 h reperfusion which were not significantly different; BBB permeability increases were associated with TJ protein reductions
Baburamani et al., 2013	E132 sheep (term = ∼145 days); hypoxia induced by umbilical cord occlusion; brain tissue collected at 24 and 48 h post-HI	Microvascular density and morphology	Umbilical cord occlusion produced a significant reduction in vascular density in the caudate nucleus, and a trend toward reduction in the cortex (*p* = 0.08) and SCWM (*p* = 0.058); occlusion produced no significant alteration in vascular morphology in any region tested
Olah et al., 2013	Newborn piglets; asphyxia induced followed by reventilation with air for 24 h, or with H_2_-supplemented air for 4 h followed by air for 20 h	Cerebrovascular reactivity of pial arterioles	Cerebrovascular reactivity to hypercapnia, NMDA was reduced at 24 h following asphyxia/reventilation; Cerebrovascular reactivity largely preserved with H_2_-supplemented air
Ek et al., 2015	P9 mice; unilateral HI brain injury induced; brain tissue, CSF, and blood samples collected at 2,6, 24 h, and 3, 7 days post-HI	BBB permeability; endothelial TJ gene/protein expression	Increased BBB permeability within 2 h of HI injury, peaking at 6 h; likely restoration of BBB function within 3 days; Reductions in TJ proteins and changes in distribution at 6 h
Diaz et al., 2016	P7 rats; unilateral HI injury induced followed by temporary exposure to hypoxia; brain tissue collected at P8, 22, and 60	BBB permeability; BBB structural protein expression	HI injury increased BBB permeability at each time point measured; BBB protein expression remained altered across the entire testing period following HI injury

*BBB, blood–brain barrier; CSF, cerebrospinal fluid; E (127), embryonic day (127); HI, hypoxia-ischemia; HIE, hypoxic-ischemic encephalopathy; KO, knockout; NMDA, N-methyl-D-aspartate; P (9), postnatal day (9); PVWM, periventricular white matter; SCWM, subcortical white matter; TJ, tight junction; WT, wild-type.*

Lee et al. (2017) reviewed BBB permeability following neonatal HI insult, with studies reviewed using a variety of models and indicators of permeability, yet raising a number of shared conclusions. Among these was a general agreement that there exists an early increase in BBB permeability, peaking 2–4 h following the initial insult (Muramatsu et al., 1997; Chen et al., 2012; Ek et al., 2015; Lee et al., 2017). Interestingly, the review noted less support in these studies for a delayed second phase of increased BBB permeability, previously documented in adult models (Başkaya et al., 1997; Lee et al., 2017). Elsewhere, however, two distinct phases of NVU dysfunction have been suggested following acute hypoxic insult. Using cerebrovascular reactivity in pial arterioles to indicate function, newborn piglets have shown a second bout of dysfunction persisting 1 day after initial HI injury, following an initial recovery in function (Olah et al., 2013). Of course, permeability and vascular reactivity represent two distinct neurovascular functions, and it is entirely possible that each displays a different response to HI injury. Likewise, although pial arterioles provide a useful indication of cerebrovascular responses to injury, as previously mentioned their generalizability to the intracerebral microvessels of the NVU is unclear.

In addition to early BBB compromise, several studies cited by Lee et al. also observed early changes in expression of endothelial TJ proteins (Chen et al., 2012; Ek et al., 2015). This was suggested as evidence of restorative mechanisms activated simultaneous to, or shortly after, BBB compromise, and helping to limit potential damage. Changes in TJ protein expression in acute hypoxic injury have been documented elsewhere (Malaeb et al., 2007), and may represent a reparative response, or it is possible that these may actually promote dysfunction, being involved in any early- or late-phase BBB permeability increases following acute hypoxic insult or reperfusion.

As well as TJ proteins, changes in endothelial transporters have also been documented in response to perinatal HI injury. Among the most well-described of these transporters is the glucose transporter GLUT1. Vannucci et al. (1996) used western blot analysis to investigate this transporter’s expression following periods of 4, 24, and 72 h recovery from unilateral HI injury in rats. Their investigation found a small bilateral increase in GLUT1 at 4 h, before the contralateral hemisphere returned to control levels at 24 h, while levels in the ipsilateral hemisphere increased substantially across the same period. By 72 h this increase was no longer present, with GLUT1 levels in both hemispheres not deviating significantly from those of controls (Vannucci et al., 1996). These findings were expanded on in a later study by the same group, which investigated GLUT1 gene expression following HI injury using *in situ* hybridization histochemistry to determine temporal changes in mRNA levels in each hemisphere (Vannucci et al., 1998). This investigation found greater GLUT1 gene expression at 1 h in the contralateral hemisphere than the ipsilateral hemisphere, before this gradually reduced such that relative gene expression in each hemisphere was consistent with levels of transporter expression documented in the previous study (Vannucci et al., 1998). Although these studies demonstrate that endothelial transporters in the cerebral microvasculature comprise part of the NVU response to acute hypoxia, GLUT1 remains just one of a vast array of transporters that may be affected. Changes in a variety of other transporters have been implicated in responses to acute hypoxia, including ion and amino acid transporters (Boado et al., 2003; O’Donnell, 2014), however, evidence for these changes in perinatal models is lacking. The interest in GLUT1 reflects its importance in both development and disease responses, however, broadening research to investigate other endothelial transporters in perinatal acute hypoxia would be beneficial in further informing our knowledge of NVU responses.

Although changes in TJ protein and transporter expression following acute hypoxia have been established, these do not represent the only proteins whose expression may be altered under these circumstances. Extracting microvessels from brain sections using laser capture microdissection microscopy has allowed for in-depth and sequential detection of protein expression patterns during post-ischemic reperfusion (Haqqani et al., 2005). Findings using this technique include variations in expression of a diverse range of proteins, including those involved in cytoskeletal and cellular integrity of vasculature, as well as ion and amino-acid transporters and pumps. This technique has also been used to demonstrate other alterations associated with post-ischemic reperfusion, including changes in expression of transcription factors and inflammatory cytokines (Haqqani et al., 2005). Importantly, the rodent models these techniques were used on were not specifically designed to represent the perinatal brain, making it difficult to generalize the results to the developing brain. Regardless, the findings offer a valuable insight into the diversity of structures that may be affected following acute hypoxic injury.

Also highlighted in the review by Lee and colleagues was a clinical study investigating neonates with HIE which also found increased BBB permeability (Kumar et al., 2008), suggesting the increased permeability seen in animal models of acute hypoxia is replicated in humans. The permeability increase observed was dose-dependent, with greater degrees of injury associated with greater leakage across the BBB (Kumar et al., 2008). Despite the suggestions such results make, the ethical challenges created by attempts to control timeframes in human studies of acute hypoxia make it impossible to determine conclusively whether the patterns of early BBB compromise seen in animal models are reflected in humans.

Studies to date support that neonatal BBB compromise appears shortly after acute hypoxic events, and possible protective mechanisms appear to be activated almost immediately, however, the damage caused by these insults can persist for much longer. Rat models have been used in one investigation to demonstrate changes in astrocytes, including hypertrophy and hyperplasia, persisting at least 3 weeks after acute hypoxic injury (Levison et al., 2001). Similarly, acute hypoxia in neonatal rats has been observed in another study to result in reduced integrity of the BBB for up to 8 weeks (Diaz et al., 2016). This is likely a result of changes in TJ protein expression, which remained altered across the same period, however, the methodology used may also have influenced the findings (Diaz et al., 2016). BBB function was assessed by measuring Evans blue dye extravasation into the brain parenchyma, with levels of extravasation observed but not quantified. The appropriateness of Evans blue dye for measuring BBB integrity has been questioned in recent years, with alternatives such as sodium fluorescein and dextrans proposed to be superior (Saunders et al., 2015). These questions are particularly pertinent to investigations into the developing BBB, where variables such as growth may increase the potential differences attributable to factors such as free dye concentrations in plasma (Saunders et al., 2015).

Several theories have been proposed to explain the mechanism by which acute hypoxia affects the BBB. It is likely that damage is not caused by any one single mechanism, instead being the combined outcome of several overlapping pathways. Endothelial TJs are likely to be the BBB constituent most susceptible to pathological conditions (Alvarez-Diaz et al., 2007), and appear to play a key role in dysfunction due to acute hypoxia. TJs represent a major functional constituent of the BBB (McConnell et al., 2017), and alterations in TJ protein expression following acute hypoxia are extensive and well documented (Mark and Davis, 2002; Brown and Davis, 2005). It is thought that a redistribution or reduction in these proteins following acute hypoxia contributes to a resultant increase in permeability (Alvarez-Diaz et al., 2007; Engelhardt et al., 2014).

Inflammatory mechanisms provide a potential basis for TJ damage following HI injury, and are implicated particularly strongly in deleterious NVU responses to acute hypoxia. Neuroinflammation is seen as a result of stroke in adults, and is a contributing mechanism to associated brain injury (Iadecola and Anrather, 2011). Inflammatory activity in the developing CNS is mediated through microglia, cells which show a dramatic upregulation following preterm HI (Jellema et al., 2013). Indeed, HI in preterm sheep has been shown to result in extensive cerebral inflammation and mobilization of the peripheral innate immune system (Jellema et al., 2013), and perinatal inflammation has been associated with BBB disruption (Nico et al., 2000; Yan et al., 2004).

Neuroinflammatory responses are complex, depending on interactions between chemokines, cytokines, reactive oxygen species, and secondary messengers (Tohidpour et al., 2017). Components of the developing NVU have displayed a particular vulnerability to these modulators. Cytokines produce BBB disruption *in vitro*, with cyclooxygenase activation in ECs appearing to play a major role (de Vries et al., 1996). The extent of free-radical injury has also been correlated with BBB permeability in newborns, providing further evidence of a causal association between inflammation and NVU damage (Kumar et al., 2008). The role of inflammatory mechanisms in causing NVU damage in acute hypoxia also provides a basis for experimental observations of beneficial responses at the BBB in response to anti-inflammatory treatments in the context of HI injury, including in the developing brain (Zhao et al., 2014; Li et al., 2015; Wu et al., 2015). The results of Wu et al. link these benefits to increases in TJ proteins and reduced cytokine expression (Wu et al., 2015), reinforcing the role of both in deleterious NVU responses to acute hypoxia.

Despite evidence supporting their role in BBB dysfunction, the complicated nature of microglial responses to perinatal HI injury is highlighted by research suggesting they may also play protective roles during this period. Newborn rodents depleted of microglia have demonstrated increased rates of hemorrhage in affected regions (Fernandez-Lopez et al., 2016), suggesting a role for microglia in microvascular stability. Such implications reinforce the immense complexity of microglial and inflammatory mechanisms, particularly in the developing brain. Although this complexity places a more detailed discussion of these mechanisms beyond the scope of this review, it is clear that this is an area warranting particular attention in future research.

Increased matrix metalloproteinase (MMP)-9 expression also has a known association with ischemia within the brain (Romanic et al., 1998; Gasche et al., 1999), with knockout mice displaying increased protection against post-ischemic BBB dysfunction and inflammation (Svedin et al., 2007). In human neonates, increased serum MMP-9 levels are seen on the day of birth following perinatal asphyxia, and appear to be correlated with the severity of neurological outcome (Sunagawa et al., 2009). Considered together, these findings strongly imply a role for MMP-9 in NVU compromise following HI injury. For example, MMP-9 has been implicated in the aforementioned endothelial TJ protein redistribution seen following HI injury (Bauer et al., 2010). Additionally, MMP-9 activation after HI injury has been correlated spatiotemporally to laminin degradation (Zalewska et al., 2002), suggesting a potential role in NVU dysfunction via BM breakdown.

#### Chronic Hypoxia

Most research to examine the association between HI insult and BBB function/dysfunction into HI injury, particularly in the developing brain, has studied insults that are acute in nature, short-lived, and involve substantial reductions in fetal and neonatal blood flow. Responses to these insults can therefore be measured in the hours and days following the initial insult. Hypoxic insults exist on a spectrum, with no specific criteria for distinguishing between acute and chronic hypoxia. Giussani’s 2016 review describes chronic fetal hypoxia as “oxygen deprivation of the unborn child lasting for several weeks or even months” (Giussani, 2016), a definition that broadly holds true for many human infants during pregnancy, particularly those in which placental function is suboptimal. Rees and Inder describe that acute hypoxic insult in late gestation is more likely to result in neuronal death and white matter injury, while chronic intrauterine hypoxia is less likely to cause neuronal loss (Rees and Inder, 2005). In light of this distinction there is a clear basis for investigating the effects of chronic hypoxia on the NVU independently of other forms of hypoxia.

Fetal cerebrovascular responses to chronic hypoxia are well documented (see Table 3). Research at altitude has identified a greater vulnerability to chronic hypoxia in the fetal cerebral vasculature than in adults (Longo et al., 1993). Elsewhere, comparisons have also found that adult changes in basilar artery vasodilation and response to K^+^ caused by chronic hypoxia are not replicated in newborns exposed to similar environments (Nauli et al., 2005). Although not based specifically on the intracerebral capillaries associated with the NVU, these results nonetheless reinforce the notion that vascular responses to chronic hypoxia differ between the perinatal and adult brain. Via effects on contractile mechanisms and receptor affinities, chronic hypoxia attenuates cerebral arterial responses to vasoactive stimuli, impeding cerebrovascular homeostatic maintenance (Pearce, 2018). Chronic hypoxia has also been linked to alterations in sympathetic perivascular innervation, causing changes in cerebrovascular smooth muscle cell differentiation, and subsequently in vascular contractility and functioning (Adeoye et al., 2015; Pearce, 2018). Fetal cerebral vasculature also undergoes remodeling in response to in utero chronic hypoxia. Cerebral arteries display increases in wall thickness mediated by hypertrophy of both ECs and vascular smooth muscle (Williams and Pearce, 2006), although once again the implications of this for the smaller, thin-walled capillaries of the NVU are unclear. Chronic hypoxia also leads to modifications in the contractile mechanisms of fetal cerebral vessels, with the result an overall reduction in cerebrovascular contractility (Pearce et al., 2011).

**TABLE 3:** Studies investigating the effects of chronic hypoxia in the perinatal period on the NVU.

**Authors**	**Experimental model**	**NVU outcome measured**	**Key findings**
Nitsos and Rees, 1990	E52 and E62 guinea pigs (term = ∼66 days); IUGR induced by SUAL at E30	Cerebral cortical mature astrocyte density	Increased proliferation of astrocytes around cerebral blood vessels following exposure to CH; no other significant difference in astrocyte development found following CH exposure
Longo et al., 1993	Near term (E139–143) sheep fetuses, maintained at an altitude of 3820 m; non-pregnant adult sheep (18–24 months) maintained in the same environment	Vascular wall thickness/inside diameter	Wall thickness not significantly altered by environmental oxygen in fetal or adult models; reduced inside diameter of arteries in adult sheep exposed to CH, with no change observed in fetal sheep
Bernstein et al., 2000	North American singleton neonates delivered between 25 and 30 weeks gestation, with birth weight between 501 and 1500 g	Rate of IVH; rate of severe IVH	Trend toward association of IUGR with increased risks of IVH (odds ratio, 1.13; 95% CI, 0.99–1.29), and severe IVH (odds ratio, 1.25; 95% CI, 0.98–1.59), although not quite statistically significant
Ogunshola et al., 2000	Newborn rats delivered naturally at term; reared under hypoxic conditions from P3 to P33; brain tissue collected at P3, 8, 13, 24, and 33	Cerebral vascular count and density; microvascular lumen diameter	Higher cerebral vascular density from P24 onward in rats exposed to CH; vascular luminal diameters significantly increased from P24 onward after exposure to CH
Gilbert and Danielsen, 2003	Californian newborns, delivered between 26 and 41 weeks gestation, and surviving to 1 year of life	Rate of IVH	IVH rate significantly lower in IUGR infants delivered 28–29 weeks than in AGA infants; IVH rates not significantly different at 30–33 weeks gestation; newborns with IUGR at increased risk of IVH between 34 and 40 weeks
Nauli et al., 2005	Near term (∼140 days) sheep fetuses, non-pregnant adult sheep (18–24 months old); maintained at altitude of 3820 m for 100 days	Contractile tension, and cytosolic [Ca^2+^] of basilar arteries removed from the brain, following administration of graded concentrations of K^+^ and serotonin	Changes in endothelium-dependent relaxation and K^+^-induced contractile tension induced by CH in adult sheep; these changes not seen in fetal sheep
Williams and Pearce, 2006	Near term (∼140 days) sheep fetuses, non-pregnant adult sheep (18–24 months old); maintained at altitude of 3820 m for 110 days	EC and vascular smooth muscle cell size and density in cerebral arteries	EC widths but not lengths reduced in both fetal and adult sheep following CH; EC density in fetal cerebral arteries increased following CH, but reduced in equivalent adult arteries; smooth muscle cell size significantly increased after CH in fetal arteries, but reduced in adult arteries
Bassan et al., 2010	E29 rabbits (term = ∼32 days); IUGR induced by uteroplacental vessel ligation at E25	Superficial cerebral cortical mature astrocyte count	Reduction in mature cortical astrocyte numbers following exposure to CH
Ortigosa Rocha et al., 2010	Singleton neonates delivered between 34 and 36+6 weeks gestation, with or without IUGR	Rate of IVH	Infants with IUGR found to be at greater risk of IVH than AGA infants
Castillo-Melendez et al., 2015	Newborn sheep delivered naturally at term (∼145 days); IUGR induced by SUAL at ∼105 days gestation	WM blood vessel density and number; vascular proliferation; pericyte and astrocyte coverage of vasculature; BBB permeability; white matter microbleeds	Vessel density and number reduced in brains of IUGR lambs; vascular proliferation reduced in IUGR lambs; pericyte and astrocyte end feet coverage reduced in IUGR lambs; signs of increased BBB permeability in IUGR lambs; microbleeds more prevalent in IUGR lambs

*AGA, appropriate for gestational age; BBB, blood–brain barrier; CH, chronic hypoxia; CI, confidence interval; E (139), embryonic day (139); EC, endothelial cell; GFAP, glial fibrillary acidic protein; H&E, hematoxylin and eosin; IUGR, intrauterine growth restriction; IVH; P (3), postnatal day (3); PECAM-1, platelet and endothelial cell adhesion molecule 1; SMA, smooth muscle actin; SUAL, single umbilical artery ligation; WM, white matter.*

##### Intrauterine growth restriction

Although intimately associated with chronic hypoxia, intrauterine growth restriction (IUGR) displays significant nutritional and endocrine involvements that distinguish it from other forms of chronic fetal hypoxia (Marsal, 2018). These distinctions make it useful to consider IUGR independently of other forms of chronic hypoxia in utero. IUGR most often results from chronic hypoxia in utero caused by placental dysfunction. Reductions in fetal growth have been demonstrated where pregnant rats have experienced long-term exposure to hypoxic environments, with growth restriction proportional to oxygen reduction (de Grauw et al., 1986). Similar effects are seen in humans, where high altitude during pregnancy has been identified as an independent risk factor for low birth weight, with a similar dose-dependent relationship observed (Jensen and Moore, 1997). Research using umbilical cord oxygen values to measure fetal oxygenation shows a strong association with infant size at birth (Lackman et al., 2001), confirming an important role for chronic hypoxia in IUGR pathogenesis. Chronic hypoxia in utero most commonly arises due to *placental insufficiency*, a general term used to describe any reduction in transfer of oxygen and nutrients from mother to fetus (Malhotra et al., 2019). This has many possible causes, including maternal hypertension or tobacco use, partial detachment of the placenta, placental villus edema, or occlusion of the uterine artery (Rees et al., 2008). By causing placental insufficiency and subsequent hypoxemia, impaired placental function provides the most significant contribution to the development of IUGR (Gagnon, 2003; Miller et al., 2016).

The fetal response to growth restriction is to preferentially redistribute blood flow toward organs considered more “essential”, including the heart and adrenals (Kamitomo et al., 1993; Poudel et al., 2015), but most prominently the brain (Damodaram et al., 2012). Consequently, this adaptive response is called “brain sparing,” and results in an asymmetric pattern of reduced fetal growth that preserves head size relative to the rest of the body (Rosenberg, 2008). Although based on a well-documented phenomenon, the term “brain sparing” is somewhat of a misnomer. Despite adaptations to provide the brain with preferential blood flow, brain development may be compromised in a number of ways. These are diverse and influenced by a range of variables, such as the timing of in utero compromise, gestational age at birth, and the existence of comorbidities (Miller et al., 2016). Among documented associations with IUGR are reductions in cortical gray matter and overall brain tissue at birth (Tolsa et al., 2004), reductions in fetal brain cell numbers (Samuelsen et al., 2007), and poor cognitive function in later life (Scherjon et al., 2000).

In addition to detrimental effects on overall brain growth, there is a growing body of evidence to demonstrate that IUGR also affects vascular development and the NVU. Castillo-Melendez et al. (2015) have highlighted the attention given to pathways of oxidative stress, excitotoxicity, and inflammation in recent research into perinatal brain injury in IUGR, lamenting the lack of focus paid to vascular responses in the developing cerebrum. In seeking to address this, they demonstrated reduced white matter vascular density, a near complete absence of EC proliferation, and loss of vascular astrocyte and pericyte attachments in the brains of IUGR neonatal lambs (Castillo-Melendez et al., 2015).

The apparent reduction in cerebral angiogenesis in response to IUGR and chronic hypoxia presents a contrast to the response of the brain to acute hypoxia, where angiogenesis is upregulated (Huang et al., 2004; Baburamani et al., 2013). Interestingly, the in utero reduction in angiogenesis also appears to differ from the postnatal response. Exposure to chronic hypoxia after birth has been found to cause an upregulation in vascular proliferation, a finding based on immunostaining targeted at PECAM-1 (Ogunshola et al., 2000). This endothelial cell marker may undergo altered expression in response to certain combinations of inflammatory cytokines (Rival et al., 1996; Stewart et al., 1996), although it is not clear whether this could affect brain tissue immunoreactivity. Studies measuring pro-angiogenic factors such as VEGF and angiopoietin-2 have demonstrated an initial increase over 1-to-2 weeks of chronic hypoxia, followed by a decline (Chavez et al., 2000; Pichiule and LaManna, 2002). Although these studies were based on adult models, a similar eventual decline in pro-angiogenic factors may explain the vascular regression observed in fetal brains exposed to chronic hypoxia. Castillo-Melendez et al. (2015) propose a reduction in VEGF-A expression combined with reduced EC proliferation as the most likely cause of the reduced vascularity seen in the developing brain in response to IUGR.

When considering the NVU, additional to an overall reduction in cerebral vascularity following IUGR is the observation of a reduction in pericyte coverage of vessels (Castillo-Melendez et al., 2015). Given the diversity of roles played by pericytes within the brain, there are a variety of adverse outcomes that might potentially result from any insufficiency. Lower pericyte density within the germinal matrix has been linked to the increased fragility and rates of hemorrhage in this region (Braun et al., 2007). It is plausible that reduced pericyte coverage seen throughout several brain regions by Castillo-Melendez et al. (2015) could result in instability of the microvascularity in these regions, and an increased rate of intracerebral hemorrhage. Indeed, the study found support for this, observing germinal matrix microbleeds in 4 of 9 IUGR lambs (Castillo-Melendez et al., 2015).

In addition to pericytes, Castillo-Melendez et al. (2015) observed an association between IUGR and a reduction in astrocyte coverage in their lamb models. Unlike pericytes, however, this reduction was not quantified and was based instead on qualitative analysis, making it somewhat difficult to interpret. Similar to the pattern observed in pericytes, the reduction in astrocyte coverage was seen across all brain areas analyzed, suggesting it is likely a global response to IUGR, at least within the developing cerebral white matter. In addition to astrocyte numbers being reduced, astrocytes that were present displayed poor contact between end-feet and blood vessels (Castillo-Melendez et al., 2015). Reduced astrocyte coverage in growth restriction is supported by recent research in a rabbit model of IUGR that showed widespread reduction in mature astrocytes throughout the developing cortex (Bassan et al., 2010). Interestingly, however, this premise is contradicted by previous research in IUGR guinea pigs, which displayed increased astrocyte proliferation around cortical blood vessels (Nitsos and Rees, 1990). This apparent discordance may potentially be a result of the disparity in timing of IUGR induction between the two studies. Bassan et al.’s (2010) rabbit model induced IUGR at E25 (term = ∼31 days), specifically targeting the end of the third trimester. Conversely, Nitsos and Rees (1990) induced IUGR in their guinea pig models at E30 (term = 66 days), representing a mid-gestation onset of IUGR. Alternatively, the disparity in outcomes may have been a result of the studies each using different animals, or a different method for inducing placental insufficiency and resultant IUGR in their models. Regardless of cause, the existence of such a profound contrast in reported findings suggests astrocyte development is a complex and multifactorial process. It also reinforces the need for further research to strengthen our understanding of the process, and particularly how IUGR and chronic hypoxia affect astrocyte morphology and function.

Similar to pericytes, astrocytes play roles in a number of brain processes, meaning any developmental alterations are likely to result in a variety of adverse effects. Astrocytes and pericytes are also implicated in several shared processes, and developmental effects targeting both cell-types are likely to produce particularly profound negative results. Castillo-Melendez et al. (2015) demonstrate at least one such example of compromised function in IUGR, with reduced BBB integrity strongly suggested through albumin extravasation from the cerebral vasculature. Unfortunately, this analysis was also qualitative, with albumin extravasation measured on its presence or absence and no mention made of the extent of leakage. This makes it difficult to determine the extent to which BBB disruption occurred in IUGR. BBB compromise in IUGR is consistent with established knowledge of the important roles both pericytes and astrocytes play in BBB function and maintenance, although we are not yet at a stage in which we understand the mechanisms by which chronic in utero hypoxia causes these cellular changes to the NVU.

Interestingly, IUGR appears also to potentially affect the risk period for IVH. Gilbert and Danielsen compared appropriate for gestational age (AGA) infants with IUGR infants born at various preterm age timepoints, and found that IUGR was associated with a lower rate of IVH in infants born between 28 and 30 weeks’ gestation (Gilbert and Danielsen, 2003). Additionally, they found no significant difference between the groups from 30 to 33 weeks, however, from 34 weeks onward IVH occurred at a significantly increased rate in IUGR infants (Gilbert and Danielsen, 2003). This is a finding supported by research comparing IUGR and AGA infants delivered between 34 and 36+6 weeks’ gestation, which also found an association between IUGR and IVH during this period (Ortigosa Rocha et al., 2010), seemingly confirming chronic hypoxia as a risk factor for IVH in late gestation. It should, however, be noted that another clinical study did not find any association between IUGR and IVH when infants were born between 27 and 32 weeks gestation, or over the period 27–35 weeks (Simchen et al., 2000). This latter study only examined cases of grade 3–4 IVH, such that any association with milder IVH cases may have been missed. Further clinical studies should aim to characterize the presence and severity of IVH in IUGR infants born preterm and at term, as this could present a cohort of infants in which altered NVU development could be targeted to improve neurodevelopmental outcomes.

## NVU Involvement in Repair and Restoration

We have presented evidence to support that NVU dysfunction due to perinatal insults is likely to contribute to poor neurological development, but we must also point out that the NVU could play a role in repair of brain injury. Within the adult brain there is a particularly strong focus on characterizing the balance between beneficial and deleterious responses to injury by the NVU (ElAli, 2016). Given the ubiquitous and highly diverse nature of the NVU components within the brain, it is perhaps unsurprising that the NVU has also been strongly implicated in repair mechanisms in response to adverse stimuli (Maki et al., 2013).

A notable beneficial role in CNS repair has been demonstrated in astrocytes, which display a particularly diverse range of phenotypes (Huang et al., 2019). Huang et al. describe several potential protective roles for astrocytes following ischemic brain injury, including alterations in metabolic pathways and mitochondrial membrane potentials to enhance neuronal survival, and involvement in intercellular mitochondrial transfer (Huang et al., 2019). Additionally, they explored astrocytic involvement in vascular function and remodeling, and immune regulation following CNS injury (Huang et al., 2019). Supportive mechanisms at the NVU do not end with astrocytes, however, with pericytes representing another component cell that can mediate restorative responses, through modification of angiogenesis, neurogenesis, and immune regulation (Geranmayeh et al., 2019). Pericytes have also been linked to mesenchymal stem cells, sharing several characteristics *in vitro*, and it has been proposed that pericytes may provide the *in vivo* source for these cells (Crisan et al., 2008; Murphy et al., 2013). When grafted, mesenchymal stem cells display substantial pro-regenerative properties (Murphy et al., 2013; Gaceb et al., 2018), and recent evidence has suggested that these properties may be shared by pericytes (Gaceb et al., 2018). Should this prove to be the case it may allow pericytes to be recruited in therapeutic efforts to mitigate the damage caused via NVU injury.

It is likely that the response of the NVU to injury represents a dynamic balance of promoting damage and repair, with the balance of these dictating outcomes (Maki et al., 2013; ElAli, 2016; Huang et al., 2019). In the context of ischemic injury, several mediators previously discussed as being implicated in NVU dysfunction actually appear to display biphasic responses, also contributing to repair mechanisms. For example, we have highlighted above the likely role of MMP-9 in acute post-ischemic NVU compromise, however, evidence also suggests that MMP-9 makes a critical contribution to the delayed phase reparative response to ischemic stroke. In adult rats, MMP-9 is upregulated 1–2 weeks post-ischemia, with inhibition during this period leading to increased injury and delayed recovery (Zhao et al., 2006, 2007). This likely reflects the role of MMP-9 in ongoing neurovascular remodeling (Xing et al., 2012; ElAli, 2016).

Presently, the signals controlling NVU outcomes are complex and remain poorly understood, particularly in the developing brain, however, they offer enormous potential as therapeutic targets. The ability to discriminate between pro-dysfunction and pro-repair signaling would theoretically allow for targeted treatments to be designed which could shift the balance of signals – and therefore outcomes – toward restorative mechanisms following ischemia. For example, using the above example of MMP-9, potential treatments might aim to inhibit early MMP-9 upregulation following acute ischemia, shifting toward a promotion of the effects of MMP-9 in later stages. Similarly, astrocytes and their signals have been identified as additional targets. One area of promise involves VEGF (expressed by astrocytes, among other cells) (Zhao and Rempe, 2010), where early post-ischemic increases have been associated with BBB leakage and hemorrhage, with latter increases promoting angiogenesis and neurological recovery (Zhang et al., 2000). Accordingly, potential therapeutics could be designed to target early inhibition of VEGF, before shifting to upregulation during its beneficial stages.

Despite the clear clinical promise displayed by protective and regenerative NVU responses to injury, these remain relatively poorly investigated in the perinatal period. Regardless, currently available evidence suggests great potential in this area, with the opportunity for translation into significant therapeutic outcomes. This potential reinforces the importance of continued NVU research as we seek to achieve these outcomes.

## Conclusion

The NVU presents a complex set of cells and interactions that are critical in regulating normal homeostatic control within the brain, and the constituent cells are highly reactive in response to adverse stimuli. Research to examine the NVU in the perinatal period has suggested significant differences between the developing brain versus adulthood, lefting this an area worthy of specific investigation. We present evidence to show that the NVU within the developing brain may be especially vulnerable to a number of insults, including prematurity, as well as acute and chronic hypoxia. Despite research that has been conducted identifying susceptibility of the NVU to perinatal insults, there remain areas of great uncertainty in our understanding of the balance of NVU responsiveness toward brain injury and repair. A better understanding of NVU response to perinatal insult would potentially guide new therapeutic options that could protect or repair the neonatal brain. We propose that the NVU presents an exceptional opportunity as a new frontier for studies in the fetal and neonatal brain, allowing investigators to make important and exciting discoveries that could impact clinical care.

## Author Contributions

AM and MC-M conceived the idea for the review. AB wrote the first draft of the manuscript. AM, MC-M, and SM edited and added to the sections of the manuscript. All authors approved the final version of the manuscript.

## Conflict of Interest

The authors declare that the research was conducted in the absence of any commercial or financial relationships that could be construed as a potential conflict of interest.
